# Protein and Polysaccharide-Based Magnetic Composite Materials for Medical Applications

**DOI:** 10.3390/ijms21010186

**Published:** 2019-12-26

**Authors:** Elizabeth J. Bealer, Kyril Kavetsky, Sierra Dutko, Samuel Lofland, Xiao Hu

**Affiliations:** 1 Department of Physics and Astronomy, Rowan University, Glassboro, NJ 08028, USA; bealere0@students.rowan.edu (E.J.B.); kavetskyk1@students.rowan.edu (K.K.); dutkos7@students.rowan.edu (S.D.); lofland@rowan.edu (S.L.); 2 Department of Biomedical Engineering, Rowan University, Glassboro, NJ 08028, USA; 3 Department of Molecular and Cellular Biosciences, Rowan University, Glassboro, NJ 08028, USA

**Keywords:** protein, polysaccharide, magnetic nanoparticles, biomaterials fabrication, nanomedicine, drug delivery, tissue regeneration

## Abstract

The combination of protein and polysaccharides with magnetic materials has been implemented in biomedical applications for decades. Proteins such as silk, collagen, and elastin and polysaccharides such as chitosan, cellulose, and alginate have been heavily used in composite biomaterials. The wide diversity in the structure of the materials including their primary monomer/amino acid sequences allow for tunable properties. Various types of these composites are highly regarded due to their biocompatible, thermal, and mechanical properties while retaining their biological characteristics. This review provides information on protein and polysaccharide materials combined with magnetic elements in the biomedical space showcasing the materials used, fabrication methods, and their subsequent applications in biomedical research.

## 1. Introduction

The use of magnetic materials in biomaterials and biomedicine has grown in popularity due to their broadening applications in the field. Particle size, surface chemistry, and composition are some of the tunable properties which allow for their integration into clinical drugs and devices [1,2]. Magnetic nanoparticles (MNPs) are often favored due to their small size and controllable magnetization [3,4,5,6,7]. MNPs are anywhere from a few nanometers to about 100 nanometers in dimension [5,7,8] and often have different properties compared to those of their bulk counterparts. In addition, magnetic materials are easily functionalized with other biomaterials such as proteins and polysaccharides that increase their biocompatibility and usefulness in applications [3,6,7,9]. These combinations of magnetic particles with proteins and polysaccharides lead to new biomaterials that can be used in various applications. One such example is tissue engineering where magnetic fields are used to promote the development of new tissue in response to damage or loss [3,9,10,11]. Another example is drug delivery where products are being tested in vivo with magnets to ultimately help release active drug therapies in patients [12,13,14,15,16].

The materials used for the aforementioned magnetic-based applications are diverse but have similar immunogenic properties that make them ideal for used as biomaterials [17,18,19,20]. Protein-based materials such as silk, keratin, and soy can be degraded in the body without adverse toxicity and have physical properties that can be tuned to the user’s needs [17,21,22]. These materials are simple to isolate and utilize enhancing their likelihood of being incorporated into various applications [23,24]. Polysaccharides (e.g., cellulose, chitosan, hyaluronan) are another group of materials that are inexpensive natural products that provide biocompatibility and diversity in application [18,25]. Their synthesis, fabrication, and structure allow for their integration with magnetics in order to be used in biomedical applications.

**Figure 1:**
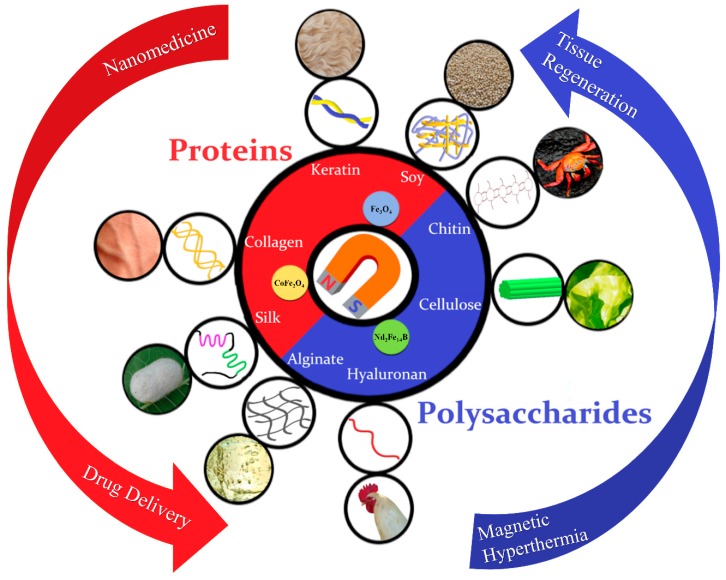
Magnetic biomaterials can be fabricated from a variety of protein and polysaccharide sources, including silk, collagen, keratin, and soy proteins, as well as polysaccharides from chitin, cellulose, hyaluronan and alginate. These natural biopolymers have different molecular structures and can be processed into magnetic composite materials with tunable properties for various biomedical applications.

This review categorizes and describes the characteristics and basic applications of various protein-based magnetic materials from animals and plants such as silks (Section 2.1.1), keratin (Section 2.1.2), soy proteins (Section 2.1.3), elastin (Section 2.1.4), and collagen (Section 2.1.5), as well as polysaccharide-based magnetic materials like cellulose (Section 2.2.1), chitin and chitosan (Section 2.2.2), hyaluronan (Section 2.2.3), and alginate (Section 2.2.4) (Figure 1). There is a discussion of the types and properties of various magnetic materials used in magnetic composites as well as a section regarding recent developments in the techniques for fabrication of such biomaterials. Lastly, we mention various applications of magnetic biomaterials such as drug delivery systems, cancer cell targeting methods, and tissue regeneration techniques.

## 2. Categories of Protein and Polysaccharide Materials

### 2.1. Protein Materials

Proteins are naturally occurring, linear, unbranched polymer chains, whose primary structure is composed of anywhere between 50 and 2000 amino acids connected to one another via peptide double bonds [26]. These series of amino acids and peptide bonds form polypeptide chains, with an ⍺-amino group or ⍺-carboxyl group. The regularly repeating part of the chain forms the backbone while the distinctive side chains vary between proteins [26]. Bonds between ⍺-carbon atoms and the amino or carbonyl group are singular and allow for freedom of rotation which permits the protein to fold in many different ways, once again avoiding steric clash [27,28].

There are two major conformations of peptides that are sterically possible and most fully exploit the hydrogen bonding available within the backbone: the ⍺ helix and the β sheet [26,29,30]. The ⍺ helix is a rod-like structure that is formed from a tightly coiled backbone with outward extending side chains. In β sheets, the polypeptide chain (β strand) is almost fully extended and the side chains of adjacent amino acids alternate directions [27,28,31]. Linking β strands by hydrogen bonding creates the β sheet. The linkage can be parallel, where the adjacent chains run in the same direction, or antiparallel, where they run in the opposite direction [27,28]. Beta sheets can be relatively flat or take on a twisted shape. The hydrogen bonds between the amine and carbonyl groups of the amino acids stabilize both the ⍺-helix and the β-sheet conformations [27,28].

The overall folding of proteins is complex and shows little to no signs of symmetry [32]. In most scenarios, the polypeptide chain folds so that the hydrophobic side chains are buried within the structure while the polar (hydrophilic) side chains are at the surface. In order for hydrophobic faces of the secondary structures (⍺ helices and β sheets) to be buried, the amine and carbonyl groups must be hydrogen bonded. The stability of this overall shape is complemented by Van Der Waals interactions between tightly packed hydrocarbon side chains [27,28].

Proteins that contain more than one polypeptide chain (subunit) display a fourth level of structural organization that is based on the spatial arrangement of each subunit and the way in which they interact with one another. Examples of these include dimers, trimers, and other quantities of identical subunits within the protein [27,28].

#### 2.1.1. Silk

Silk is a fibrous protein produced by animals from moths and butterflies to spiders and scorpions [33,34,35,36]. Its hydrophobic side chains, consisting mainly of glycine, alanine, and serine, allow for the formation of β sheets within the protein and give silk its notoriously high tensile strength. The hydrophilic amino acids in the structure of silk provide its elasticity [17,37,38]. Crystallinity of the β sheet can be tuned by methanol treatments in order to modify its mechanical properties [39,40]. The most extensively studied and utilized silk comes from the domestic silkworm, *Bombyx mori*, due to its large-scale production and its biocompatibility. The cocoon of the *Bombyx mori* is made from naturally multi-layered silk fibroin fibers that display a unique hierarchical structure which leads to its specific and singular strength and toughness [17]. Silk from the *Nephila clavipes* spider has also been popular in recent studies due to its ability to promote cell adhesion and migration. Both silkworm and spider silks have proven to be promising biopolymers in the biomaterials industry as tissue scaffolds [17].

#### 2.1.2. Keratin

Keratins are cysteine-rich fibrous structural biopolymers that are the major components of hair, feathers, nails and horns [41]. Two α helices wind around one another to form its superhelix structure; one helix is designated type I, the other type II. There are two distinct groups of these proteins based on the amino acid sequence: hard—short crystalline fibers embedded in a highly cross-linked elastomeric matrix (hair and nails); and soft—cytoskeletal elements found in epithelial tissues [41,42]. Hard keratins have large amounts of cysteines that interact through disulfide bonds which give them their enhanced mechanical strength compared to that of soft keratins. Both groups have intrinsic biocompatibility, biodegradability, mechanical durability, and natural abundance, making keratins very popular in studies on their biomaterial applications [22,42,43].

#### 2.1.3. Soy

Soy is a plant-based protein that is obtained from the soybean plant (*Glycine max* L. Merr.) [44]. This plant is comprised of a heterogeneous assortment of proteins, mainly globular storage proteins with two main subunits, conglycinin 7S and glycinin 11S [44]. These subunits both contain non-polar amino acids: alanine, valine, and leucine; basic amino acids: lysine and arginine; and non-charged polar amino acids: cysteine and glycine [17]. With the exception of the sulphur-containing amino acids, soy protein mimics the amino acid patterns of high-quality animal protein sources [44,45]. Soy protein is highly biodegradable and environmentally friendly, [46] and it is relatively easy to acquire soy due to its abundance in nature. Soybean products such as soy protein isolate is used as an adhesive in industrial applications [17]. Its biocompatibility allows for its use in tissue engineered scaffolds and as a wound dressing [46].

#### 2.1.4. Elastin

Elastin is an extracellular matrix protein that provides elasticity to tissues and organs such as ligaments, lungs, blood vessels and arteries [47]. It consists of alternating hydrophobic and hydrophilic domains, wherein the hydrophilic domains there are lysines scattered among alanines. This allows for the cross-linkage of the molecules, which is further strengthened by the repetition of the hydrophobic glycines, valines, and prolines [17]. Tropoelastin, the in vivo precursor to elastin, has been targeted for its use in regenerative medicine. It crosslinks and has a direct impact of the structure and flexibility on the protein [48]. Elastin protein has a resistance to enzymatic, chemical and physical degradation which suggests long-term stability in biomedical or biomaterial applications [49,50]. In addition to stability, its self-assembly behavior, elasticity, and biological activity make elastin-based biomaterials greatly desirable [49,50,51,52,53,54].

#### 2.1.5. Collagen and Gelatin

Collagen is the most abundant protein in animals, presenting in the skin and tendons, cartilage, bone, and most internal organs. It is a fibrous and structural protein that provides structural and mechanical support [49,51,55,56] and also aids in tissue functions such as scaffolding, morphogenesis, and repair [57]. There are nearly 30 different types of collagen that have been identified and the three main types that can be found in the human body are classified as Type I, II, or III. Every type of collagen contains a repeating amino acid sequence where two amino acids are followed by a glycine. This allows for a stable three-stranded α helical structure to form. Type I collagen is the most naturally abundant and is used the most frequently in biomaterial studies [17]. Collagen is a good matrix material for tissue engineering due to its ability to act as a natural substrate for cell attachment and allows for the development of bioengineered tissue scaffolds [17,58]. Gelatin is derived from collagen hydrolysis and is a biodegradable, bioresorbable material [59,60,61]. It is found as a mixture of peptides and type A (acid hydrolysis) and type B (alkaline hydrolysis) are the two gelatins that are usually formulated from native collagen [60]. These biomaterials are favorable due to their low cost and biocompatibility [59,62]. Recently the chemical and physical structure of gelatin has led to its use as a drug carrier agent and in cell imaging applications [62].

### 2.2. Polysaccharide Materials

Polysaccharides are relatively complex carbohydrates that are formed from ten or more repeating monosaccharides or disaccharides [63,64]. They are generally more stable than proteins since they are usually not irreversibly denatured when heated [65]. Additionally, polysaccharides possess linear or branched structure and can be monofunctional or polyfunctional. Other beneficial properties include their high levels of chirality, water solubility or insolubility, and low toxicity [18,64]. There are storage (starch and glycogen) and structural (cellulose and chitin) polysaccharides. Most polysaccharide-based biomaterials are derived from structural polysaccharides, and their use in the biomedicine field is expanding.

#### 2.2.1. Cellulose

Cellulose is a structural polysaccharide that is contained within the cell walls of plants and animals [66,67]. Long polymer chains of glucose units connected by β-acetal linkages form its crystalline structure, where β-glucose molecules join together to create a polymer [67]. Due to the intramolecular hydrogen bonding within the joined glucose, cellulose can form very rigid fibers [68]. Different types of cellulose can be formed through unique methods, such as treating natural cellulose (cellulose I) with sodium hydroxide, soaking it in cold (approximately −80 °C) liquid anhydrous ammonia, or soaking it in hot (approximately 200 °C) glycerol. These methods transform cellulose I into a crystalline matrix with a different set of properties. Of these, cellulose II (made by treating cellulose I with sodium hydroxide) is the most thermodynamically stable [18]. The aforementioned properties such as stability in temperature and mechanical properties allow for the fabrication of cellulose.

#### 2.2.2. Chitin and Chitosan

Chitin is a long, linear chain of N-acetyl-d-glucosamine residues that naturally forms highly crystalline ordered networks. The arrangement of chains in chitin can be designated as α, β, and ɣ chitin. α-chitin is mostly found in arthropods and crustaceans, and has chains in a parallel arrangement; β-chitin has an antiparallel arrangement; and ɣ-chitin is a combination of parallel and antiparallel where out of every three chitin chains, two are parallel and one is antiparallel [18,69]. Chitin is widely distributed among plants and animals and even found in the cell wall of fungi and algae. Its strong intermolecular interactions through hydrogen bonding improve the rigidity of chitin and limit its solubility.

Chitosan is also a linear polysaccharide composed of β-(1,4)-linked D-glucosamine and is obtained by extensive deacetylation of chitin. Unlike chitin, chitosan is soluble in dilute aqueous solutions. It has many properties that make it favorable to the biomedical community, such as its bioactivity, antimicrobial activity, immunostimulation, chemotactic action, enzymatic biodegradability, mucoadhesion and epithelial permeability [70,71].

#### 2.2.3. Hyaluronan

Hyaluronan (hyaluronic acid) is a simple, water-soluble polysaccharide [72] whose structure is an unbranched glycosaminoglycan chain that is formed from repeating disaccharide units containing D-glucuronic acid and N-acetyl-d-glucosamine. It is expressed in the extracellular matrix, on the cell surface, and inside the cell where it functions to maintain tissue hydration and lubrication [73]. It is also found in soft connective tissues, joints, skin, eyes, and other tissue-derived organs. Hyaluronan is important in homeostasis of plasma proteins and is thought to have a role in the mitotic process due to its ability to help cells proliferate [74].

Hyaluronan has unique hydrophilic, rheological, and viscoelastic properties that contribute to its significant role in biological processes and make it a good biomaterial [75]. The matrix of this polysaccharide allows for cells to be in a hydrated environment and can increase their development [75,76]. Applications in biomedicine to date include use as a serum for eye surgery and various other inflammatory conditions [74].

#### 2.2.4. Alginate

Alginates are carboxylic polysaccharides that are produced almost exclusively in brown algae. Their major components are the L-guluronate and D-mannuronate residues, whose ratio varies depending on the natural source and form linear copolymers [77,78,79]. It is believed that only the α-L-guluronate residues participate in intermolecular cross-linkage in order to form hydrogels; therefore, the guluronate/mannuronate ratio, overall sequence, and length of the guluronate blocks are the driving factors affecting the physical properties of alginate and its hydrogels [77,78,79]. They have a thickening characteristic that allows them to increase the solvent viscosity upon dissolution, thus allowing for physical stabilization within the solution. Their ionic change aptitude and gel-forming ability in the presence of multivalent counterions is a direct consequence of alginates being polyelectrolytes and is why they follow the usual behavior of charged polymers. When in a fibrous or film form, alginate is used as hemostatic materials and wound dressings [72].

## 3. Magnetic Properties of Materials for Biomedical Applications

### 3.1. Magnetism of Materials for Composites

Table 1 lists typical magnetic particles used today in various medical applications. The defining characteristic of a magnetic material is the magnetization **M**, the net magnetic moment per unit volume and is dependent on the magnetic field **H**. The particular response depends upon the type of magnetic material for which there is great diversity, including diamagnetic, antiferromagnetic, ferromagnetic, ferrimagnetic, paramagnetic, and superparamagnetic phases. Since most of the nanocomposites rely upon maximizing the magnetic forces, this discussion will focus on ferromagnetic and superparamagnetic materials.

**Figure 2:**
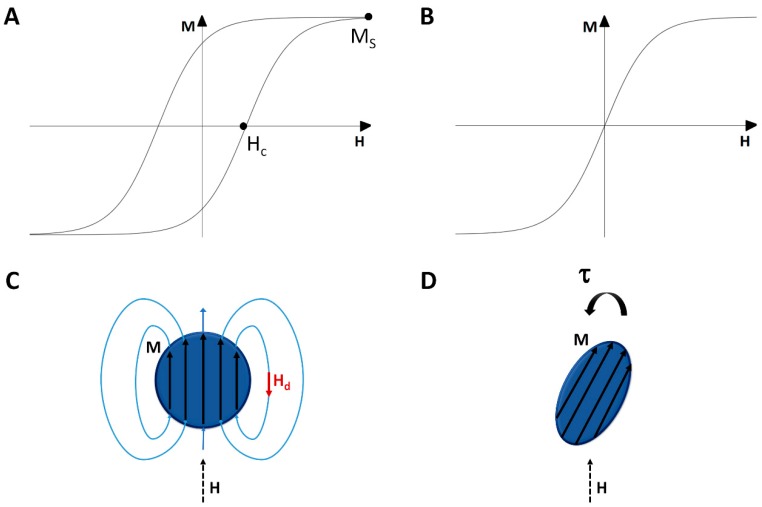
Schematic *M-H* curves of (**A**) A ferromagnet and (**B**) A superparamagnet; (**C**) A schematic showing the demagnetization field in a spherical magnetic particle; (**D**) A schematic showing a non-spherical magnetic particle can experience a magnetic torque even in a uniform field.

For ferromagnetic materials, there exists a spontaneous magnetization which exists in the absence of an applied field. This is due to the interaction between the spins of neighboring *d* and *f* shell elements which align parallel to each other in order to minimize the electrostatic energy. Because of competition between various contributions to the energy (anisotropy, demagnetization, Zeeman, exchange), there are local energy minima which generally give rise to hysteretic *M-H* curves (Figure 2A,B). At sufficiently high values of *H*, *M* approaches the saturation magnetization *M_s_*. On reducing the field, a remanent magnetization persists at zero applied field and only when the magnetic field opposes the magnetization with a magnitude of *H_c_*, the coercive field, does *M* change sign.

Many of the magnetic materials used in composites are nanoparticles of ferromagnetic materials; however, their small size alters their magnetic properties from that of a bulk material as first discussed by Néel [93]. The strong exchange interaction ensures that all spins are aligned with each other but the magnetic anisotropy which gives rise to a preferred magnetic orientation is much weaker and scales with the volume. When the particles are small enough, the thermal energy can be larger than the anisotropy energy, and so the magnetization direction can wander. Such a system is called superparamagnetic in that it acts like a paramagnet with noninteracting moments that align with *H* except that instead of the moment being that of a particular ion, it is the moment of an entire particle. Such superparamagnetic systems are generally anhysteretic and have a large susceptibility:

*χ* = d*M*/d*H* (1)

### 3.2. Shape Effects of Magnetic Particles

For any finite size object, there are dipolar fields which emanate from the object due to the magnetization. Since the magnetic field lines always form closed loops, this demagnetization field *H_d_* opposes the magnetization (Figure 2C). The actual magnetic field

*H* = *H*_app_ + *H_d_* (2)

where

*H_d_* = −*NM* (3)

and *H*_app_ is the applied magnetic field and *N* the demagnetization factor. For spherical particles, *N* = 1/3. For long rods, *N* = 0 along the axis and *N* = 1/2 in the transverse direction. For the special case of ellipsoids, the magnetization is uniform inside, and the values for *N* have been worked out by Osborn [94].

### 3.3. Forces and Torques on a Magnetic Sample

The magnetic energy density *u* is given by

*u* = −µ_0_**M**⋅**H** (4)

where µ_0_ is the permeability of vacuum. Since most particles are often approximately spherical or spheroidal in shape, **M** is uniform throughout the particle if **H** is uniform, which is a reasonable approximation given that the gradient in **H** will have a much larger length scale than the submicron scale of the particle. However, if the particle density is sufficiently large, this assumption may break down due to dipolar fields from neighboring particles. Note that it is these dipolar fields which generally contribute to the contrast enhancement in MRI by magnetic nanoparticles.

The force **F** on a given magnetic particle is given by negative the gradient of the potential energy *U*, and if we assume that the density is small enough to ignore particle-particle interactions, then

**F** = −∇*U* = −µ_0_*V*∇(**M**⋅**H**) ≈ µ_0_*V*(**M**⋅∇)**H** (5)

where *V* is the volume of the particle. The rightmost equation is true because ∇ × **H** = 0 since there are no currents in the sample and because **M** is nearly uniform. In essence, the force is dependent on the product of the **M** and the gradient in **H** although one needs to keep in mind that **M** itself is dependent on **H**.

The magnetic torque density **τ** is given by

**τ** = µ_0_**M** × **H** (6)

If *H*_app_ is not along a principle axis of the object, then **H** and **M** are not parallel. Thus, magnetic particles, even in a uniform field, can experience magnetic torques (Figure 2D). The magnetic torque has a tendency to align the axis of the object with the smallest value of *N* to the magnetic field. This means that spherical particles experience no torque while needles tend to orient with their primary axes along **H**.

## 4. Fabrication Methods

### 4.1. Electrospinning

**Figure 3:**
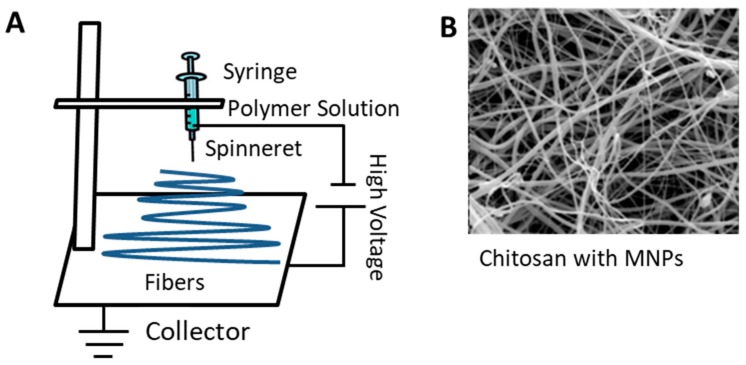
(**A**) Common vertical set-up for electrospinning, along with (**B**) an example of chitosan nanofibers with MNPs (Scale in B: 100 nm). (B is reproduced from open access Ref [122], De Gruyter, 2017).

Table 2 summarizes advantages, parameters and applications of common magnetic material fabrication methods based on proteins or polysaccharides. Electrospinning is a unique technique for fabricating protein or polysaccharide-based magnetic materials. The process of electrospinning was developed over one hundred years ago [95,96] and uses electrostatic forces to fabricate thin, nanoscale fibers. A syringe pump, DC power supply, and ground collector are the primary components of an electrospinning system (Figure 3) [96,97,98,99,100,101]. An electric potential of about 15~20 kV is needed to maintain the process and is used in the two main conventional setups, vertical and horizontal [102]. The methods allow for different ways to collect the fibers, and the process itself is constantly being updated to form fibers with varied orientations [103,104]. When the potential is applied, charge induction will form what is known as a Taylor cone due to surface tension and electrical force [96,97]. When the electrical force exceeds the capillary pressure, fibers are drawn from a droplet that forms at the top of a spinneret [96] and guided to the ground collector [105,106,107].

There are a number of factors that affect the fiber properties including the applied voltage, temperature, and humidity, among others. Normally, electrospinning is done at room temperature, and the resultant fibers often range in size from nanometers to microns [108,109,110,111]. Many types of fibers can be produced such as polymer, composite, and synthetic which have led to electrospinning’s use in a variety of fields [96,100,112,113].

Electrospun fibers with magnetic particles can be used in a variety of biomedical applications that include tissue engineering [114,115] and drug delivery [116,117]. The success of the former is because the nanofibers have high surface areas and therefore promote adhesion, proliferation, and differentiation of cells [118,119]. Magnetic composite fibers have been synthesized by adding the magnetic nanoparticles to the solution prior to processing the fibers [84]. Cobalt ferrite (CoFe_2_O_4_) and magnetite (Fe_3_O_4_) are commonly used magnetic nanoparticles, and their presence can alter the physical properties of the fibers. [7,84,120,121].

### 4.2. Film Casting

**Figure 4:**
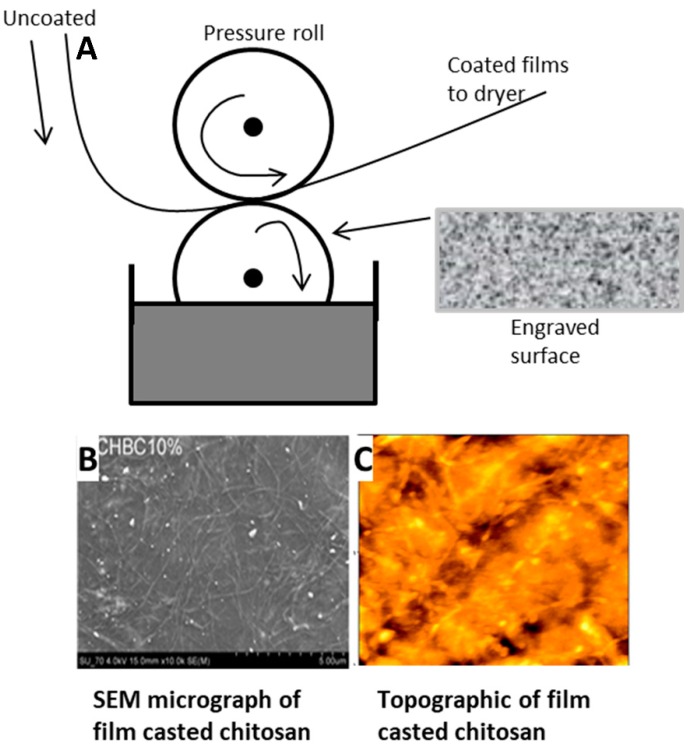
(**A**) Example of the set-up for the film casting process. Seen below (**B**,**C**) are two examples of films casted that are made from chitosan. (**B**) is an SEM micrograph (Scale 100 μm) and (**C**) is a topographic image of the surface of the film. ((**B**,**C**) are reproduced with permission from Ref [127], Copyright RSC Publishing, 2009).

Thin sheets or films can be produced via the film-casting method which is shown schematically in Figure 4 [123,124,125]. This fabrication method is similar to electrospinning but differs in the direction that the film/sheet is drawn; this process stretches the film/sheet planarly. The molten solution in a solvent is added to a flat-surfaced moving belt. The solution will spread out, cool, and dry on the belt, completely removing the solvent, and the dry film can then be removed [125]. This method can produce films or sheets anywhere from about ten to a few thousand microns in thickness and with large widths of up to a few hundred centimeters [124,126]. The speed of the film casting process determines the ratio of the film thickness to its width and influences the physical properties [124].

Film casting is often used to prepare nanocomposites [18]. with magnetic and polysaccharide-based materials having been used. Cellulose and chitosan are examples of polysaccharides that are used in film casting that have strong thermal stability [127,128]. Copper and gold particles that were degassed have been used to produce films that possess magnetic properties [129]. Film casting has been used to modulate the antimicrobial properties, transparency, and thermally conductivity [18] of composite biomaterials.

### 4.3. Dip Coating

The dip-coating process has been commercially in use since 1939 [130]. It is a relatively simple process that has been since used for thin films and sol-gel technology. Dip coating involves immersion of a substrate in the desired solution to create a layer of the solution on the substrate [131,132,133]. This method utilizes chemical interactions such as hydrophobic effects and electrostatic and ionic interactions. In general, the process is split into five steps: immersion, startup (substrate remains in solution), deposition, drainage, and evaporation [133]. This process can be beneficial for objects that are irregular or complex in shape [125,132]. Though it was primarily done by hand at first, the automation of dip coating has been used commercially to enhance reproducibility. The benefits of dip coating also include an easily adjustable thickness layer, convenience, and low cost; however one downside is the possible blocking of the “screen” of the substrate which can change the product’s function [130,131].

There are multiple factors that influence dip coating results such as the rate of condensation and evaporation, capillary pressure, and withdrawal speed [134,135]. The film thickness specifically is determined by capillary force and gravity [134,135]. Other factors in the process are viscous drag, surface tension [131,132] and density [136].

**Figure 5:**
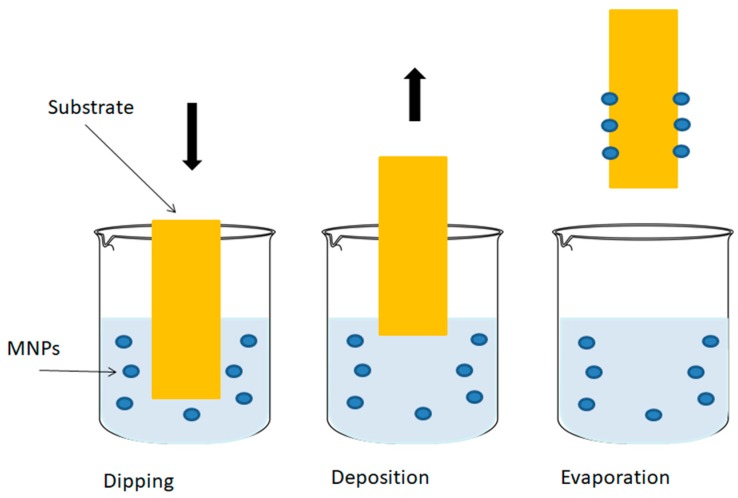
Schematic of the dip coating process showing the dipping, deposition of MNPs and evaporation to create the protein or polysaccharide magnetic material that can be used in biomedical applications.

Dip coating can be used to synthesize magnetic nanocomposites with a protein or polysaccharide matrix (Figure 5) [18]. A dip-coated composite of silver and chitosan has demonstrated enhanced thermal properties for antimicrobial applications [137]. Dip-coating techniques have also been considered allow for use in therapeutic biomedical applications such as tissue engineering [18,138].

### 4.4. Infusion Gyration

**Figure 6:**
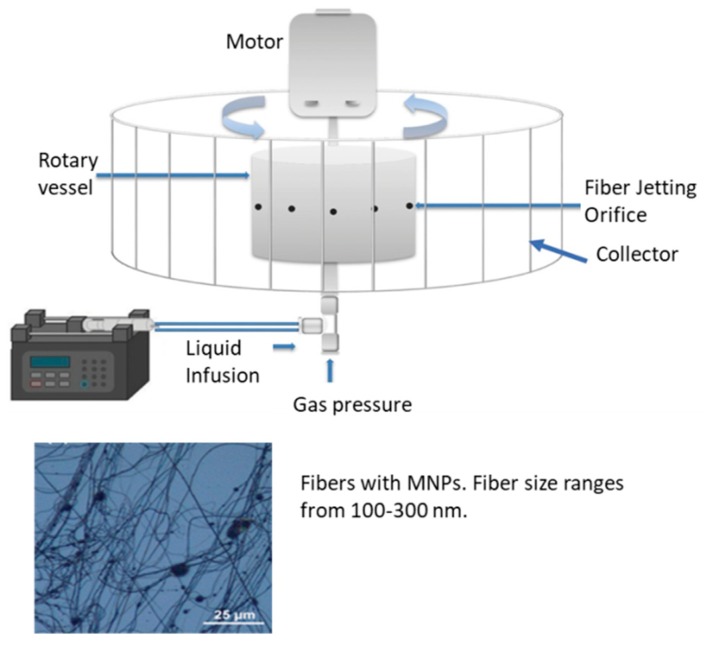
Infusion gyration method of fabrication and examples of fibers created from this method. For example, these fibers are fabricated with the addition of Fe particles at a speed of 36,000 rpm [14,142] (Scale bar is 25 μm). (Reproduced with permission from Refs [14,142], ref. [142]: Copyright John Wiley and Sons, 2017; ref. [14]: American Chemical Society, 2018, further permissions related to the material excerpted should be directed to the ACS).

Infusion gyration is another process that has been developed to fabricate fibers [14,139,140,141]. Seen in Figure 6, the device has a hollow aluminum vessel with small holes. The system is powered by a DC motor and its rotational speed is controlled [14,139]. A syringe pump controls the flow into the spinning vessel, and fibers are collected on aluminum foil sheets in the container [14,139]. Differences in fiber size and size distribution can be attributed to the shape and volume of the polymer droplets flow at different rates at the opening of the apparatus [139]. The fibers that are created are also impacted by the concentration and viscosity of the solution that is infused into the vessel [102,140].

This method comes from a pressurized gyration technique [140] that helped Zhang et al. develop the Berry number (Be) to represent fiber size. In their experiment a Be score of less than 1.6 would not result in nanofibers because of low polymer chain entanglement while a score of above 4.8 resulted in a thick fiber. They came to the conclusion that speed for infusion gyration should be around 36,000 rpm for fiber formation [139].

The flow rate can be adjusted to tune the properties of the fibers to engineer smooth and well-aligned fibers. This method has been used to fabricate protein nanofibers which have potential biomedical applications due to their biocompatibility. Subsequently, magnetic composite nanofibers have been synthesized and for candidates as drug-release agents and as materials for imaging [14,139].

## 5. Recent Magnetic Applications of Protein and Polysaccharide Materials

### 5.1. Tissue Regeneration

The applications of tissue engineering are expanding rapidly with great promise to create diverse new tissues to treat various human conditions [3,9,10,11,148,149]. Magnetic nanoparticles and magnetic cationic liposomes are currently being implemented in force-based tissue engineering [3,150,151] while some tissue engineered constructs have been coated with a mixture of iron oxide particles and collagen [10]. These studies demonstrate that magnetic scaffolds can help improve adhesion and proliferation of various progenys of cells including bone marrow, bone, adipose, cartilage, and tendon while remaining biocompatible [10,24,152].

**Figure 7:**
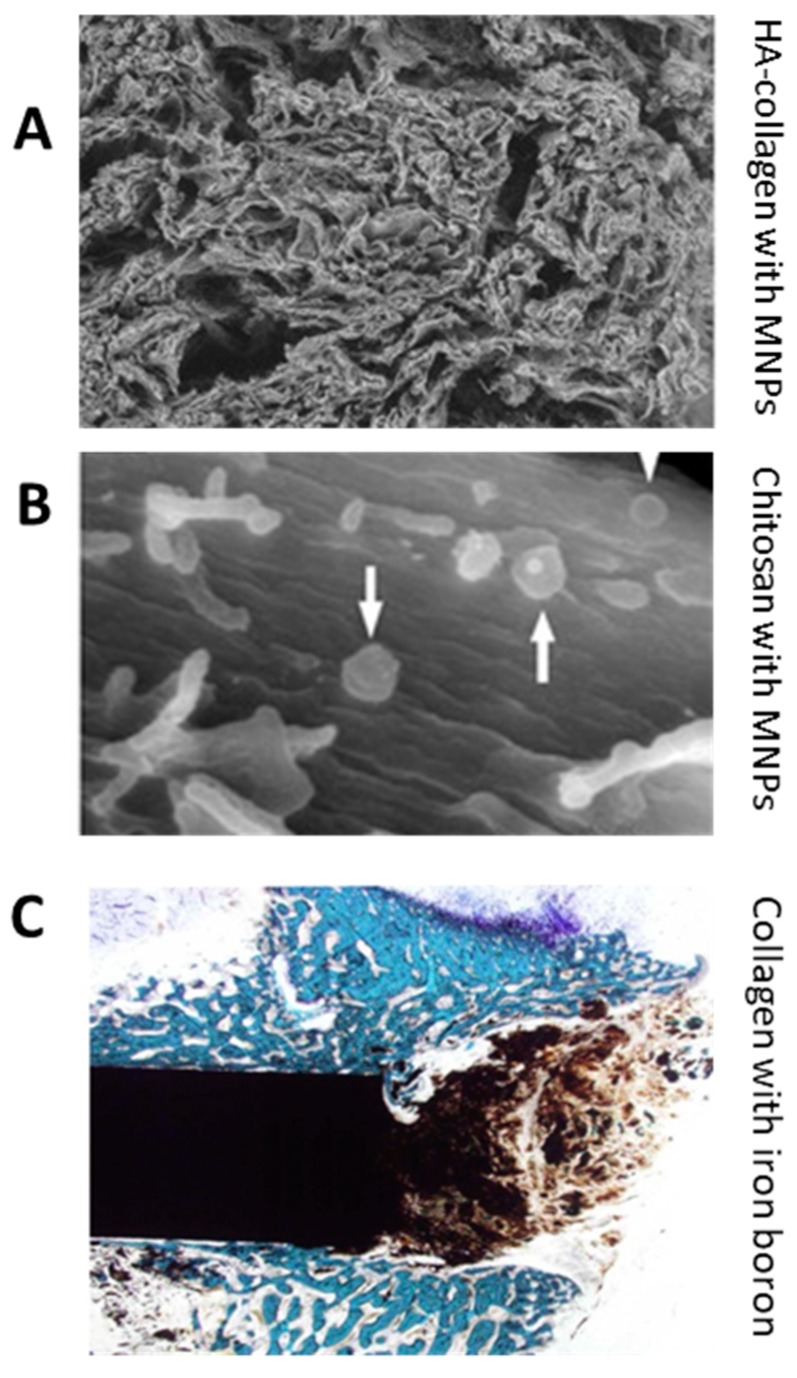
Examples of tissue engineered scaffolds with protein or polysaccharide magnetic combinations. (**A**) Hydroxyapatite-collagen scaffold with magnetic nanoparticles dip coated on them (image width: 2500 µm). (**B**) Magnetic nanoparticles with chitosan. The white arrows show the MNPs and this image shows cells seeded on the scaffold (image width: 3.84 µm). (**C**) A NdFeB magnet placed in bone showing influence of magnet on cells (image width: 6.39 mm). (Reproduced with permission from Refs [10,91,153], (**A**) Copyright Elsevier, 2010; (**B**) Elsevier, 2007; (**C**) John Wiley and Sons, 2013).

The combination of chitosan and magnetic material was investigated by Sasaki et al. [153] to show its abilities in the tissue engineering and regenerative medicine space (Figure 7A). The magnetic nanoparticles were coated with chitosan and then added into cells. Cylindrical neodymium magnets were used in this study. This enhanced the cell seeding in 3D scaffolds—A crucial issue in tissue engineering. Cells often do not penetrate the tissue engineering construct and will remain superficial. The magnetic force was able to push the seed cells deeper into the scaffolds. The degree of magnetic force directly impacted how effective the invasion was. Cell-to-cell interactions and proliferation times were improved with MNPs. The chitosan aided in the biocompatibility and biodegradability of the MNPs as well. Cells were approximately 80% viable after the addition compared to 100% of the control. This study showed promise and the hope for future designs that will lead to more cell infiltration in tissue engineering.

Bock et al. [10] presented another application of magnetic scaffolds that used hydroxyapatite and collagen (Figure 7B). There scaffolds were fabricated by dip-coating methods that added ferrofluids with iron oxide particles. They concluded that their scaffolds should be further looked at for tissue engineering applications. The proliferation of human bone marrow stem cells in vitro was achieved. The magnetic nanoparticles were also able to be guided in the scaffold. This did not affect the scaffold, which is essential to keep the porosity and shape of the construct [10].

Another study of collagen and MNPs was done by Panseri et al. [91] (Figure 7C). These scaffolds were in vivo and had external magnets that helped to control regeneration. The orientation of the scaffold was affected by the magnetics and the ECM was deposited similar to the orientation [89,91,154]. A NdFeB magnet was used and created a static magnetic field that augmented the bone tissue engineering. The magnets were able to “fix” the scaffold and reduce the need for pin fixation that is commonly used in bones. The overall goal in this study is to help with engineering of osseous structures like metaphyseal bone [89].

Tissue engineering will be a large focus of protein and polysaccharide magnetic materials for years to come. There are many applications that are still being investigated to promote proliferation and adhesion of cells in vivo. The magnetic composites have shown to respond to magnetic fields along with the ability to move cells and elements (e.g., growth factors) of the scaffold while the protein and polysaccharide materials allow for better biocompatibility. This combination of tissue constructs could ultimately lead to greater control over cell behavior and expansion.

### 5.2. Drug Delivery

Studies have shown that protein and polysaccharide composite magnetic materials are advantageous for drug delivery due to their timed release [12,13,14,15,16,155,156,157,158]. There are efforts to use these materials to produce targeting systems where the magnetic portion of the biomaterial can be guided to a specific site in vivo [12,15,159,160,161]. Drug-laden silk fibroin magnetic nanoparticles have been used in cancer research to access tumor sites [138,162]. Various polysaccharides such as heparin and chitosan have been used with superparamagnetic iron oxides in the delivery of anticancer drugs and for tumor tagging capabilities [18,80,83].

**Figure 8:**
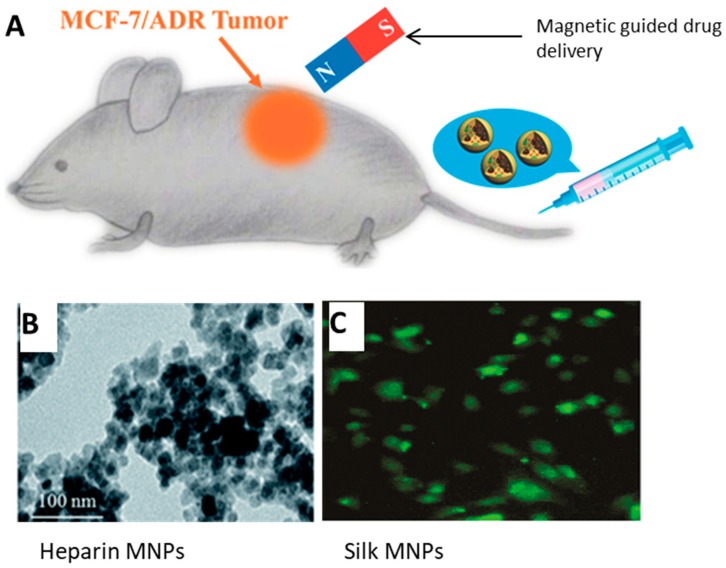
Drug delivery uses of magnetic nanoparticles and protein/polysaccharide materials. (**A**) The full drug delivery system used by Tian et al. that shows the combination of MNPs and silk protein. (**B**) Images of a heparin-coated iron-oxide magnetic nanocomposite (Scale: 100 nm). (**C**) Composite of MNPs and silk to deliver curcumin. (Reproduced with permission from Refs [80,162,163] (**A**) Copyright ACS, 2017; (**B**) John Wiley and Sons, 2014; (**C**) RSC Publishing, 2014).

Tian et al. [162] presented a novel cancer drug therapy with silk fibroin and magnetic nanoparticles (Figure 8A). Their process was to salt out silk fibroin with doxorubicin (DOX) and magnetite MNPs. The combined particles were tested on a human breast adenocarcinoma cell line (MCF-7 cells) and its resistant counterpart (MCF-7/ADR cells). An external magnetic field was applied to guide the drug to these resistant tumors in mice. According to their results, the tumors grew more extensively when magnets were not attached to the drug-loaded silk fibroin while the silk fibroin helped to protect the mice from the toxic effects of the chemotherapeutic drug. This study demonstrated in vivo tumor targeting and drug delivery that could be effective against multidrug resistant forms of cancer using the synergistic properties of MNPs and silk fibroin composite.

Another study on drug delivery was done by Javid et al. [80] using heparin-coated superparamagnetic iron oxide nanocomposites (Figure 8B). They used DOX and paclitaxel (PTX) within the core of the nanocomposites with A2780 and OVCAR-3 human ovarian cancer cells. The results showed DOX and PTX being released steadily with the application of a magnetic field, and the heparin allowed for higher uptake of the drugs along with great suppression of tumor growth. Doses of 10 μg mL^−1^ caused apoptosis in the tested cells at rates above 75%. The authors concluded that the tumor cells were internalizing the heparin-coated loaded nanocomposites.

Song et al. [163] used silk fibroin in conjunction with MNPs to deliver curcumin (Figure 8C), an anticancer drug has limitations such as low bioavailability, poor solubility, and short half-life. The goal of this study was to improve the effects of the drug with the introduction of the magnetic silk fibroin core. A breast adenocarcinoma cell line (MDA-MB-231cells) was tested. The nanoparticles could be modified by changing the silk and curcumin concentrations which affected the zeta potential, loading efficiency, and release rate. It was found that the drug could be made to be released over a period of up to 20 days, and the nanoparticles were able to inhibit growth of the cancer cells.

In another drug delivery system, gelatin was used with Fe_3_O_4_ nanoparticles [164]. The particles were shown to be distributed throughout the matrix and the applied magnetic fields increased the release rate of vitamin B-12 [164]. Gelatin and magnetic silica mesoporous nanoparticles were used to deliver paclitaxel [165]. These results showed some preliminary anti-tumor efficacy with the use of an external magnet [165].

Drug delivery methods that have been fabricated with magnetic materials and both proteins and polysaccharides have been growing in magnitude considerably in the past decade. Magnetic composite materials have been shown to be able to reduce toxicity of the drug and be able to target specific ligands. The addition of biomaterials and magnetics help to bring forth future applications that allow for more efficacious drug delivery and hopeful stronger therapeutics in common, debilitating diseases such as cancer.

### 5.3. Nanomedicine and Other Biomedical Applications

Magnetic resonance imaging (MRI) was one of the first implementations of magnetism into the biomedical field and has since been expanded to include various aspects of medicine and nanomedicine [83,166]. Alginate has been introduced for use in MRI to aid in cell tracking and to act as a negative control contrast agent [81,82,83,167]. Cellular therapies and labeling are now being implemented with MNPs in conjunction with polysaccharides and proteins to improve medicine [168,169,170,171,172,173,174,175].

**Figure 9:**
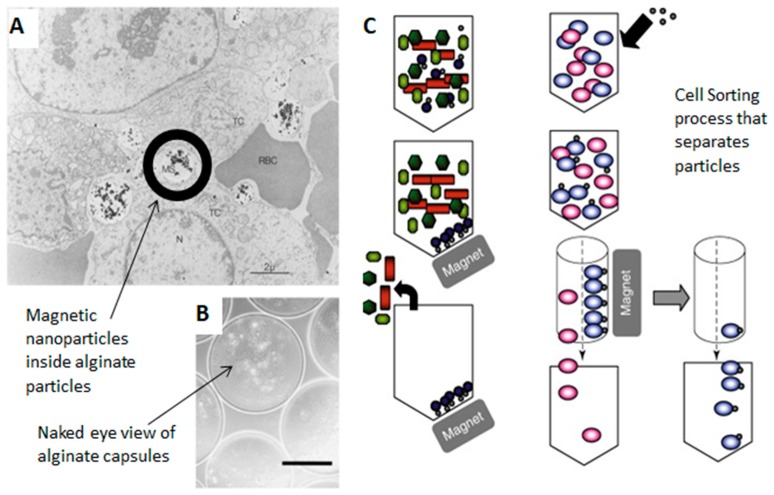
Applications of magnetics with proteins and polysaccharides in nanomedicine. (**A**) TEM image of a tumor with magnetic particles in the interstitial space of the tumor (Scale bar: 2 µm). (**B**) Particles with ferrofluid in the core of alginate capsules. These are light microscope images (Scale bar: 200 µm) (**C**) Cell sorting process that separates particles with magnets. (Reproduced with permission from Refs [81,151,168], (**A**) Copyright Elsevier, 2003; (**B**) John Wiley and Sons, 1998; (**C**) Taylor & Francis, 1998).

Shen et al. [81,82] have described a way to use alginate microcapsules for MRI (Figure 9B). The microcapsules contain ferrofluid made from iron oxide and alginate and can be accessed without surgery. The ferrofluid microcapsules can be seen with MRI because of a reduction in the *T*_2_ relaxation time which increases the contrast, making it visible compared to the alginate alone. This can be used for in vivo tracking of microcapsules to see the effects in the body in real time.

Magnetic nanoparticles have also been used in protein purification. The addition of MNPs into the process allowed for high-resolution separations without the need for centrifugation, membrane separation, or filtration. An example of this was shown by Chiang et al. [176] who used phosphopeptides MNPs, and the tagged fusion proteins could be extracted from *E. coli* with high yields [151,176].

Targeting is the effort to locate evasive cancer cells which can then be tagged in vivo. This has been another active area of investigation for magnetic nanoparticles and proteins/polysaccharides. In a study done by Pulfer and Gallo [168] with magnetic aminodextran microspheres, they were able to locate glioma tumors in rats (Figure 9C). The particles were able to remain in the brain for extended time periods and the administration of the magnetic field could change the tissue distribution. Daldrup-Link et al. [169,170] were able to label human hematopoietic progenitor cells with magnetic polysaccharide nanoparticles that could change the relaxation rate and thus change the MRI imaging. Signal intensities were improved with the addition of these contrast agents, providing another way to locate cells in the body and tag them.

Cancer cell death and the shrinking of solid tumors have been enhanced with the use of magnetic hyperthermia in recent studies [177,178,179,180]. The temperature is raised to 40–43 °C and the effects of chemotherapy are inceased with the induction of heat [179]. Cell viability and protein denaturation are affected by even a raise in a half of degree [179]. Chang et al. [178] used uniform magnetic nanoparticles of iron oxide and coated it with human-like collagen protein. An alternating magnetic field was able to raise the temperature faster than non-collagen-coated nanoparticles. This application was able to increase biocompatibility with a protein coating that could lead to further use of these composites for magnetic hyperthermia in the future.

Nanoparticles will continue to be used in various biomedical applications. Though tissue engineering and drug delivery are the most commonly used, targeting and MRI are some other examples of the diverse power of MNPs with proteins and polysaccharides. It is expected that these materials can be used in microgel assembly and mimic cell functions as the technology continues to develop [3,181].

## 6. Conclusions

The use of magnetic material with protein and polysaccharides has enhanced numerous biomedical applications in the past decade. MNPs used in fibers and microspheres have been shown promise in tissue engineering, drug delivery, and nanomedicine. For example, the ability to guide proteins and polysaccharides by magnets can be an effective therapeutic to deliver cancer drugs. Multiple methods allow for the integration of magnetic and biomedical materials. Proteins such as collagen, elastin, keratin, soy, and silk serve as biocompatible, adaptable materials to be used in these aforementioned applications. Polysaccharides including cellulose, chitin, chitosan, hyaluronan, and alginate offer further thermal stability and antimicrobial properties. These materials can also be augmented during fabrication to create layers or coatings with MNPs. Electrospinning, film-casting, dip-coating, and infusion-gyration techniques have been successfully done in studies to create these composite materials. These methods can be tuned based on the materials used to change product elements such as fiber thickness and coating concentration. Ultimately the introduction of the magnets and protein/polysaccharides allow for effective cell proliferation for tissue engineering, guidance and delivery of drugs, and tagging in imaging.

## Figures and Tables

**Table 1:** Common medical applications of magnetic particles used today.

	Structure	Composite Materials	Example Biomedical Applications
Magnetite (Fe_3_O_4_)	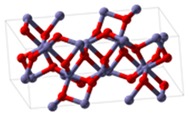 cubic spinel	Iron oxide particles and collagen [10] Iron oxides and chitosan [18] Heparin-coated superparamagnetic iron oxide nanocomposites [80] Microcapsules made from iron oxide and alginate [81,82]	Inclusions in tissue scaffolds to increase cell proliferation [10] Drug delivery and tumor targeting methods [18,80,83].
Cobalt ferrite (CoFe_2_O_4_)	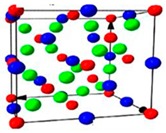 tetragonal spinel	Cobalt ferrite and silk fibroin [84] Cobalt ferrite alginate composite beads [85] Zwitterionic chitosan shell and cobalt ferrite [86]	Hyperthermia treatment Magnetic resonance imaging Magnetic separation Drug delivery systems [87,88].
Neodymum iron boron (Nd_2_Fe_14_B)	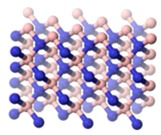 Tetragonal	Neodymium iron boron magnet with collagen [89] Neodymium iron boron magnet with chitosan and the inclusion of iron oxide particles [90]	Static magnetic field altering bone engineering [91] Enhancement of cellular uptake of superparamagnetic nanoparticles in a delivery system [92].

**Table 2:** Advantages, parameters, and applications of the common protein or polysaccharide based magnetic material fabrication methods. Source [14,18,84,108,109,110,111,118,119,124,125,126,127,128,132,134,135,136,137,139].

	Composite Composition	Advantages	Parameters Affecting Process	Applications
Electro-spinning	Chitosan and iron (II) and iron (III) salts [122] Chitin and iron chloride [143] Cellulose acetate and silver nanoparticles [144] Cobalt ferrite, iron oxide and silk fibroin [84]	Fabrication of nanosized particles Large surface area per mass Small pore size Tunable properties to make rigid or flexible fibers	Extruded solution Applied voltage Temperature Humidity	Tissue engineering Promotion of cell adhesion
Film Casting	Cellulose and chitosan with gold nanoparticles [127,128] Iron oxide and chitosan [145]	Wide range of sizes (up to a few thousand microns) Implementation in manufacturing Strong thermal stability	Speed of process Aspect ratio (distance stretched over width) Temperature	Tissue engineering constructs
Dip Coating	Silver and chitosan [137] Iron oxide and collagen [146] Heparin and silver nanoparticles [18]	Adjustable thickness Low cost and convenience Applicability for irregularly shaped objects Automation	Rates of condensation and evaporation Viscous drag Surface tension Viscosity and density of coating material Rate of withdrawal speed	Drug delivery Antimicrobial surfaces
Infusion Gyration	Lysozome protein and gold nanoparticles [141] DsRed-AuBP2-engineered protein and silver nanoparticles [147]	Flow control Control of fiber size Smooth, well-aligned fibers	Concentration and viscosity of solution infused Berry number Speed of rotation	Magnetic actuation Drug release
